# Vine Foliar Treatments at Veraison and Post-Veraison with Methyl Jasmonate Enhanced Aromatic, Phenolic and Nitrogen Composition of Tempranillo Blanco Grapes

**DOI:** 10.3390/foods12061142

**Published:** 2023-03-08

**Authors:** Itziar Sáenz de Urturi, Freud M. Ribeiro-Gomes, Sandra Marín-San Román, Rebeca Murillo-Peña, Lesly Torres-Díaz, Miriam González-Lázaro, Eva P. Pérez-Álvarez, Teresa Garde-Cerdán

**Affiliations:** Grupo VIENAP, Instituto de Ciencias de la Vid y del Vino (CSIC, Gobierno de La Rioja, Universidad de La Rioja), Ctra. de Burgos Km. 6, 26007 Logroño, Spain

**Keywords:** white grape variety, elicitor, phenolic compounds, amino acids, volatile compounds, *Vitis vinifera*

## Abstract

Methyl jasmonate (MeJ) is an elicitor that, when applied in the vineyard, can improve grape quality. There are several studies about the MeJ influence on red grape varieties; however, to our knowledge, there is little information about white grape varieties, specifically Tempranillo Blanco. Therefore, the aim of this work is to evaluate the effect of MeJ foliar treatments, carried out at veraison and post-veraison, on the aromatic, phenolic and nitrogen composition of Tempranillo Blanco grapes. The results showed that grape volatile compounds content increased after MeJ application, especially terpenoids, C_13_ norisoprenoids, benzenoids and alcohols, and, in general, mainly at post-veraison. Regarding phenolic and nitrogen compounds, their concentrations were enhanced after MeJ treatments, regardless of application time. Consequently, MeJ treatment improved grape volatile, phenolic and nitrogen composition, particularly when this elicitor was applied post-veraison. Therefore, this is a good and easy tool to modulate white grape quality.

## 1. Introduction

Tempranillo Blanco is a mutation of the Tempranillo grape variety that was discovered in Murillo de Río Leza (La Rioja, Spain) in 1988 [1]. This variety was authorised in the D.O.Ca. Rioja in 2008 and is currently one of the white grape varieties with a larger area under cultivation. This variety has similarities with Tempranillo, such as morphological characteristics and adaptation behaviour to growing conditions in La Rioja or response to diseases and pests [2]. Tempranillo Blanco, compared to other white grape varieties, has a high content of organic acids and a high concentration of phenolic compounds [3].

Due to the effects of climate change, vine physiology and phenology are changing, resulting in a mismatch between phenolic and technological maturities [4,5], which is conditioning grape and wine quality, increasing the concentration of sugars in the berry and causing a decrease in acidity without reaching optimum phenolic maturity. Currently, to mitigate these effects, some techniques are being used to improve the phenolic content in grapes, such as cluster thinning or deficit irrigation [6]. In this regard, there is a growing interest in the use of elicitors since they are molecules capable of activating plant defence mechanisms, contributing to their resistance against external attacks. Preliminary studies have shown that foliar application of methyl jasmonate (MeJ) can affect grape composition, mainly phenolic compounds [7,8,9], nitrogen compounds [10,11,12] and volatile compounds [13,14,15]. All of these studies have been carried out with red grape varieties. There are only two works where foliar treatment with MeJ has been performed in white varieties, focused on the effect on the terpene content in grapes [16] and in a white table grape variety, in which the influence of MeJ on phenolic composition was studied [17]. MeJ foliar application is an interesting and easy viticultural practice to improve the grape’s aromatic, phenolic and nitrogen composition. In addition, this work allows us to evaluate the behaviour of an important white grape variety in La Rioja and in one so little studied as Tempranillo Blanco.

Grapes are rich in many secondary metabolites, such as phenolic compounds, which are classified into two main groups: flavonoids (anthocyanins, flavonols and flavanols) and non-flavonoids (hydroxybenzoic acids, hydroxycinnamic acids and stilbenes). The concentration of phenolic compounds is conditioned, among other factors, by soil characteristics, climatic conditions, environmental stress and grape variety. Phenolic compounds play a fundamental role in grape and wine quality as they are responsible for sensory attributes such as colour, astringency or bitterness [8]. In addition, they play an important role as plant protectors against biotic and abiotic stress factors. Phenolic compounds stand out for their health-promoting properties due to their antioxidant activity [18]. Preliminary studies showed an increase in polyphenol concentration after foliar treatment with MeJ [7].

The nitrogen composition of the grape largely determines the quality of the wine, as amino acids are precursors of important fermentative volatile compounds [19]. In addition, the amount of nitrogen influences the growth and development of yeasts; hence, to ensure proper vinification and avoid stuck fermentation, the minimum assimilable nitrogen content is approximately 140 mg N/L [20]. However, an excess of nitrogen can have negative consequences, with the formation of undesirable compounds [21]. Nitrogen composition depends on multiple factors such as growing conditions, terroir, fertilisation and grape variety [22]. It should be noted that the application of chemical fungicides reduces the concentration of amino acids in grapes [23]; nevertheless, Garde-Cerdán et al. [10,24] reported that the foliar application of nitrogen compounds can improve the concentration of amino acids in the musts.

Therefore, the quality of must and wines is determined by several parameters. One of the most important is the grape’s aromatic composition [25]. Within the volatile compounds of the grape, varietal and pre-fermentative aromas can differentiate [26]. Previous studies show an increase in volatile compounds after MeJ application in grape varieties, such as Garnacha [27], especially terpenoids and C_13_ norisoprenoids.

There are many studies in reference to Tempranillo but not to Tempranillo Blanco; a study of the phenolic, nitrogen and aromatic profiles of the grapes of this white variety is needed. In view of all the foregoing, the aim of this work is to study the effect of the foliar application of an elicitor, such as MeJ, to improve the phenolic, aromatic and nitrogen composition of grapes. For this purpose, two partial objectives were established: to analyse the impact of the foliar application of methyl jasmonate on the phenolic, nitrogen and volatile composition of the Tempranillo Blanco variety and to determine the most appropriate time to apply the treatment (veraison or post-veraison).

## 2. Materials and Methods

### 2.1. Vineyard, Treatments and Grape Samples

The Tempranillo Blanco (*Vitis vinifera* L.) variety grown in the experimental vineyard located in Finca La Grajera, Logroño, La Rioja (Spain) (42°26′26″ North Latitude; 2°30′51″ West Longitude, at 447 m above sea level) in the 2020 season was used. Climatic data were obtained from the Agroclimatic Information Service of La Rioja (SIAR); the weather station was located near the plot. Annual precipitation was 498 L/m^2^, with the accumulated precipitation from bud breaking to harvest (April to August) of 190 L/m^2^ (38% of annual precipitation). Over the growing season (April to August), the average maximum temperature was 25.1 °C, and the average minimum temperature was 13.3 °C.

The vineyard was planted in 2002 with a spacing between rows of 3.00 m and within the rows of 1.10 m. Grapevines were grafted onto 110-Richter rootstock, and a training system was used in the vertical shoot position. The soil was classified as *Typic haploxerepts*. The texture is loamy in the two most superficial horizons (55 and 70 cm) and sandy-loam in the subsurface horizon (13 cm). The soil had no physical–chemical or nutritional limitations.

Methyl jasmonate (MeJ) foliar applications to the vineyard were studied at two phenological stages: veraison (MeJ-Ver) (EL: 34–37; BBCH: 83–85) and post-veraison (MeJ-Post), i.e., seven days after veraison. To carry out the foliar applications, aqueous solutions were prepared with a concentration of 10 mM of methyl jasmonate (MeJ) (Sigma-Aldrich, Madrid, Spain), using Tween 80 (Sigma-Aldrich) as the wetting agent (1 mL/L), according to previous works [15,28]. Control plants were treated with Tween 80 water solution. All treatments were applied to the grapevine twice at veraison or post-veraison and one week later (DOY MeJ-Ver, first application: 215; DOY MeJ-Ver, second application and MeJ-Post first application: 222; DOY MeJ-Post second application: 229). For each application, 200 mL/plant was sprayed over leaves. The treatments were performed in triplicate and were arranged in a complete randomised block design, with three vines for each treatment and replication.

Grapes from all grapevines and treatments were picked at their optimum technological maturity when the potential alcoholic strength of the grapes reached 13% (*v/v*). A random set of 140 berries per replicate and treatment was collected and frozen at −20 °C until the analyses of volatile (50 berries), phenolic (50 berries) and nitrogen (40 berries) composition were carried out. Another set of 100 berries was separated and weighed to obtain the weight of 100 berries. Then, grape berries were crushed, and general parameters were determined in the different musts.

### 2.2. Determination of General Parameters in Musts

The must enological parameters were analysed by OIV [29] official methods: ºBrix, probable alcohol, pH and total acidity. In addition, glucose, fructose, tartaric and malic acids, total phenols, amino nitrogen, ammonium nitrogen and yeast assimilable nitrogen (YAN) were determined using a Miura One enzymatic instrument (TDI, Barcelona, Spain).

As the treatments were performed in triplicate, the results of these enological parameters are shown as the average of 3 analyses (*n* = 3).

### 2.3. Analysis of Must Volatile Composition by HS-SPME-GC-MS

The determination of volatile compounds in the musts was carried out by headspace solid-phase micro-extraction (HS-SPME) and subsequent analysis by gas chromatography (GC) coupled to mass spectrometry (MS), according to the method described by Garde-Cerdán et al. [30]. The SPME fibre used was divinylbenzene/carboxen/polydimethylsiloxane (DVB/CAR/PDMS, 50/30 µm) (Supelco, Bellenfonte, PA, USA). In 20 mL vials (Supelco), 9 mL of the sample, 2.5 g NaCl and 10 µL of 2-octanol (internal standard) were added. After adding a stir bar, the vial was closed and placed in the GC-MS (Agilent, Palo Alto, CA, USA). Sample conditioning was done at 60 °C for 15 min and with stirring (500 rpm). After this step, the fibre was automatically inserted into the headspace for the extraction of the must volatile compounds for 105 min with agitation (500 rpm). After completion of the extraction process, the fibre was immediately inserted into the GC injection port at 250 °C and held for 15 min for desorption of the aromatic compounds. The capillary column used was SPB™-20 (30 m × 0.25 mm I.D. × 0.25 μm film thickness) (Supelco). Helium was used as the carrier gas at a flow of 1.2 mL/min. The chromatographic conditions used were: initial temperature, 40 °C for 5 min, a temperature gradient of 2 °C/min, up to a final temperature of 220 °C, to be maintained for 20 min (total time = 115 min). The ionisation of the volatile compounds was performed at 70 eV. The detector worked at full scan mode (35–300 *m/z*). Identification was carried out using the NIST library and compared with the mass spectra and retention times of chromatographic standards (Sigma-Aldrich), when available, as well as with data found in the literature. Semi-quantification was performed, relating the areas of each compound to the area and known concentration of the internal standard (2-octanol).

Since the treatments were performed in triplicate, the results of volatile compounds in musts are expressed as the mean of 3 replicates (*n* = 3).

### 2.4. Determination of Grape Phenolic Composition by HPLC-DAD

#### 2.4.1. Extraction of Grape Phenolic Compounds

Phenolic compounds were extracted from 50 g of grape samples with 50 mL of an extractant solution of methanol/water/formic acid (50:48.5:1.5, *v/v/v*) according to the method reported by Portu et al. [9]. The grapes were homogenised using an Ultra-Turrax T-18 (IKA, Staufen, Germany) at 18,000 rpm for 1 min, producing a homogeneous paste. Then, the extraction of phenolic compounds was carried out for 10 min in an ultrasonic bath (JP Selecta, Barcelona, Spain). After 10 min, the samples were centrifuged at 5000 rpm for 10 min at 10 °C (Centrifuge 5810-REppendorf, Hamburg, Germany). The supernatant was collected, and the resulting pellet was second-extracted. Finally, the two supernatants were mixed, and the volume obtained was recorded. Samples were frozen at −20 °C in 250 mL amber plastic bottles for the subsequent determination of phenolic compounds by HPLC-DAD.

#### 2.4.2. Analysis of Grape Phenolic Compounds by HPLC-DAD

Phenolic compounds were separated, identified and quantified from grape extracts by high-performance liquid chromatography (HPLC) using an Agilent 1260 Infinity chromatograph coupled to a diode array detector (DAD). The chromatographic conditions were based on the Castillo-Muñoz et al. [31] method. Samples were filtered with a 0.45 μm filter (OlimPeak, Teknokroma, Barcelona, Spain), and the separation was performed on a reverse-phase column (LiCrospher 100 RP-18; 250 × 4.0 mm I.D.; 5 μm particle diameter; Agilent), with a LiCrospher 100 RP-18 precolumn (4 × 4 mm; 5 μm particle size, Agilent) thermostated at 40 °C. For the analysis of phenolic compounds, the injection volume was 20 μL, and the flow rate was 0.630 mL/min.

Phenolic compounds were identified according to the retention times of pure compound standards (Sigma-Aldrich). For the quantification of phenolic compounds, DAD chromatograms were extracted at the following wavelengths for each chemical family: 360 nm (flavonols), 320 nm (hydroxybenzoic and hydroxycinnamic acids and stilbenes) and 280 nm (flavanols). Moreover, the calibration graphs of the respective standards (R^2^ > 0.988) were done for each family of phenolic compounds. Quercetin-3-O-glucoside was used for flavonols; *trans*-caftaric acid was used for hydroxycinnamic acids; gallic acid was used for hydroxybenzoic acids; catechin was used for flavanols; *trans*-piceid and *trans*-resveratrol were used for stilbenes.

As the foliar applications were performed in triplicate, the results for grape phenolic compounds correspond to the average of 3 analyses (*n* = 3).

### 2.5. Analysis of Must Nitrogen Composition by HPLC-DAD-FLD

The separation, identification and quantification of amino acids were carried out by HPLC using an Agilent 1260 Infinity Series coupled to a DAD and a fluorescence detector (FLD). Amino acid analysis was performed by the method described by Garde-Cerdán et al. [10]. Sample preparation was made by homogenising 40 berries in a Masticator homogenisator (IUL Basic, Barcelona, Spain). Then, the samples were centrifuged at 4000 rpm for 10 min and 20 °C. To 5 mL of each must sample, 100 μL of sarcosine (internal standard to quantify proline) and 100 μL de norvalina (internal standard to quantify primary amino acids) were added. The mixture was filtered through a 0.45 μm filter (OlimPeak) and submitted to automatic derivatisation with o-phthaldialdehyde (OPA Reagent, Agilent) for primary amino acids, with 9-fluorenylmethylchloroformate (FMOC Reagent, Agilent) for proline, the secondary amino acid. The injected volume was 10 μL, and a constant column temperature of 40 °C was maintained. All separations were performed on a column, Hypersil ODS (250 × 4.0 mm, I.D. 5 μm, Agilent). The eluents that were used as mobile phases were: A: 75 mM sodium acetate and 0.018% triethylamine (pH 6.9) + 0.3% tetrahydrofuran; B: water, methanol and acetonitrile (10:45:45, *v/v/v*).

The identification of the amino acids was made by comparison with the retention times of the standards of each amino acid (Sigma-Aldrich) as well as the UV–vis spectral characteristics. Their quantification was carried out using the calibration graphs of each respective standard (R^2^ > 0.96). DAD at two wavelengths (λ = 338 nm for primary amino acids; λ = 262 nm for the secondary amino acid, proline) and FLD (λ excitation = 340 nm, λ emission = 450 nm, for primary amino acids; λ excitation = 266 nm, λ emission = 305 nm, for the secondary amino acid, proline) were used for the detection.

Since the treatments were performed in triplicate, the results of the must nitrogen compounds are expressed as the mean of the 3 replicates (*n* = 3).

### 2.6. Statistical Analyses

The statistical study was performed using the SPSS statistical package (Chicago, IL, USA). General enological parameters and the volatile, phenolic and nitrogen compounds data were processed using the variance analysis (ANOVA) (*p* ≤ 0.05).

## 3. Results and Discussion

### 3.1. General Parameters in the Musts

Table 1 shows the parameters in control grapes and in samples from MeJ-treated vines at veraison (MeJ-Ver) and post-veraison (MeJ-Post). The weight of 100 berries increased with MeJ application at veraison, with respect to post-veraison. Although neither of the two treatments showed significant differences with the control for this parameter, it seems that the application of MeJ at veraison may favour vineyard production. Moreover, glucose concentration was higher in the control samples compared to MeJ post-veraison samples, with intermediate values for MeJ-Ver grapes. This may be related to the ripening delay found by D’Onofrio et al. [14] in their Sangiovese grapes after MeJ application. On the other hand, ammonium nitrogen and YAN increased after foliar application of MeJ-Ver, with intermediate values for MeJ-Post grapes. In addition, amino nitrogen increased after foliar application of MeJ, regardless of the time of elicitor application. These nitrogen content increases in the treated grapes could be related to the elicitor effect of MeJ since it activates the plant’s enzymatic metabolism. It was also observed that all samples reached the minimum recommended YAN content of approximately 140 mg N/L to achieve a correct development of alcoholic fermentation [20]. For the rest of the general parameters, no significant differences were observed due to the foliar application of MeJ (Table 1).

### 3.2. Influence of the Foliar MeJ Treatments on Must Volatile Compounds

**Figure 1:**
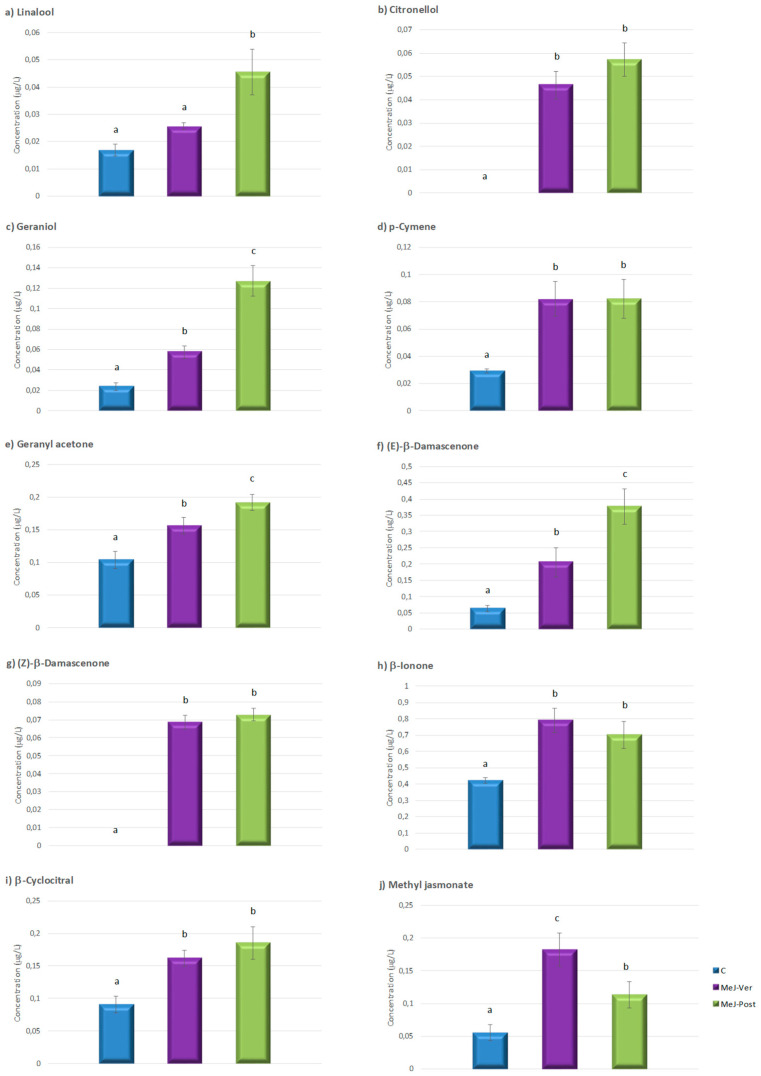
Terpenoid and C_13_ norisoprenoid concentrations (μg/L) in musts from control and MeJ treatments at veraison (MeJ-Ver) and post-veraison (MeJ-Post). All parameters are listed with their standard deviation (*n* = 3). For each compound, different letters indicate significant differences between samples (*p* ≤ 0.05).

**Figure 2:**
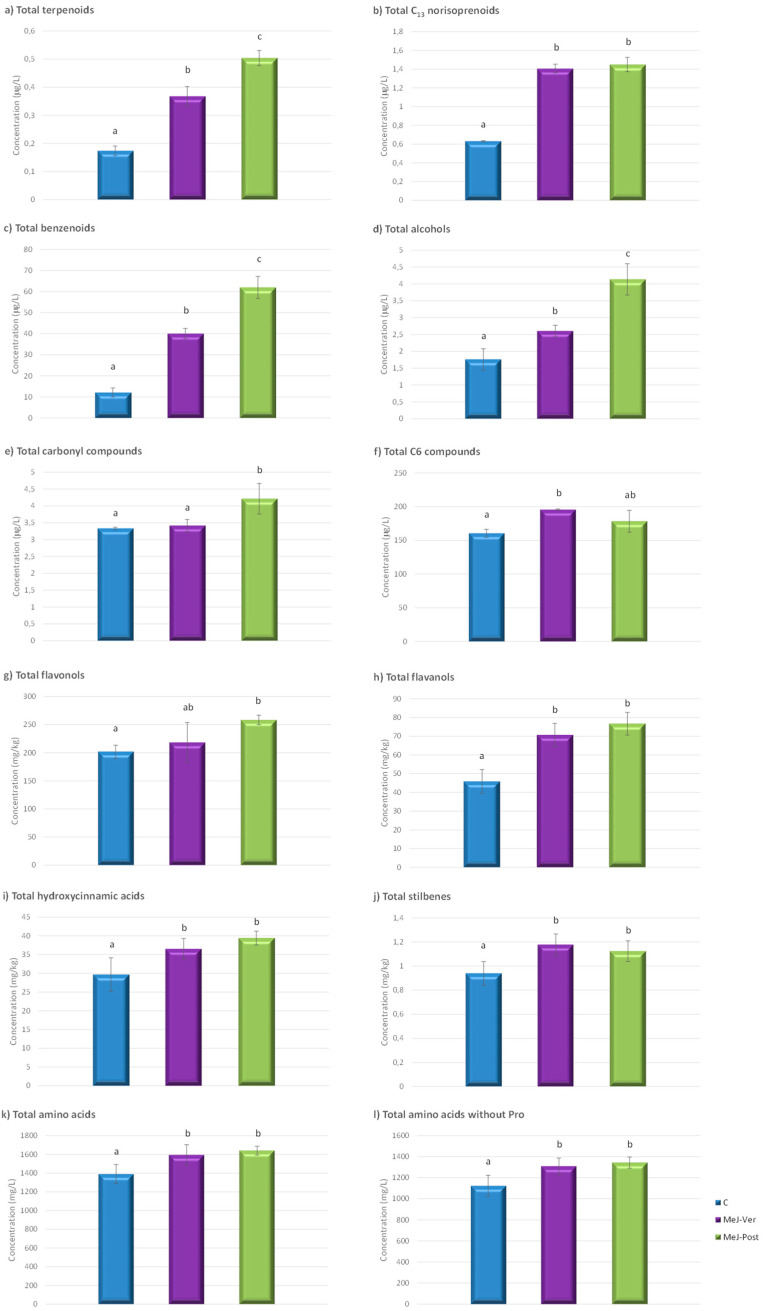
Total concentration of the different chemical families of volatile (μg/L), phenolic (mg/kg) and nitrogen (mg/L) compounds in the samples from control and MeJ treatments at veraison (MeJ-Ver) and post-veraison (MeJ-Post). All parameters are listed with their standard deviation (*n* = 3). For each compound, different letters indicate significant differences between samples (*p* ≤ 0.05).

Figure 1 and Figure 2 and Table 2 show the results of the must volatile primary aroma content in the control and in the samples from the treated grapevines with MeJ at veraison (MeJ-Ver) and post-veraison (MeJ-Post).

Within the group of terpenoids, linalool, citronellol, geraniol, *p*-cymene and geranyl acetone were identified (Figure 1a–e); however, citronellol was not found in the control samples (Figure 1b). For all compounds, an increase in their concentration was clearly observed in the samples treated foliarly with MeJ, with the exception of linalool for MeJ-Ver (Figure 1a). In the case of citronellol and *p*-cymene, this increase was significant regardless of the time of application (Figure 1b,d). In this regard, for geraniol and geranyl acetone (Figure 1c,e), as well as for total terpenoids (Figure 2a), the highest concentration was observed in the samples treated after veraison (MeJ-Post). Yue et al. [32] also observed an increase in this group of compounds with the application of this elicitor in the vineyard since it regulates their synthesis and activates their de novo biosynthesis. This increase, described in the terpene content when applying MeJ, both individually (Figure 1a–e) and totally (Figure 2a), is very important from the organoleptic quality point of view since these compounds have low thresholds of olfactory perception and contribute decisively to the fruity and floral aroma [33].

In the group of C_13_ norisoprenoids, (E)-β-damascenone, (Z)-β-damascenone, β-ionone, β-cyclocitral and methyl jasmonate were identified (Figure 1f–j); (Z)-β-damascenone was not found in the control samples (Figure 1g). For (Z)-β-damascenone, β-ionone, β-cyclocitral and total C_13_ norisoprenoids, an increase in their concentration was observed with the foliar application of MeJ, regardless of the time of application (Figure 1g–i and 2b). However, for (E)-β-damascenone and methyl jasmonate (Figure 1f,j), the effect of the elicitor application was different, depending on the application time; in the case of methyl jasmonate (Figure 1j), the highest concentration was found at veraison (MeJ-Ver), whereas, for (E)-β-damascenone, the highest concentration was observed in the MeJ-Post samples (Figure 1f). Marín-San Román et al. [27] also found an increase in this family of volatile compounds with the application of MeJ, probably due to the fact that MeJ increases the activity of the enzymes involved in the synthesis of these compounds [34]. As mentioned for terpenes, C_13_ norisoprenoids are also of great importance for aroma, likewise due to their low perception thresholds, which make their contribution to aroma relevant and important as they confer floral notes [35]. Therefore, the treatment with MeJ, either at veraison or at post-veraison, is an effective technique in order to improve the aromatic quality of Tempranillo Blanco.

Three benzenoid compounds, 2-phenylethanol, 2-phenylethanal and benzyl alcohol, were identified in the control and MeJ-treated samples (Table 2). For all of them, the treatment with MeJ increased their concentration. It should be noted that the samples treated foliarly with MeJ at post-veraison reached the highest concentration for each of the benzenoid compounds identified (Table 2) as well as for the total content of these compounds (Figure 2c). It should be highlighted that these compounds are positive for aroma, so applying MeJ at post-veraison could be a good tool to increase their content in grapes. A positive effect of the application of MeJ on the content of these compounds was also observed in the Tempranillo variety, a variety from which Tempranillo Blanco comes by spontaneous mutation, as has been mentioned. However, a foliar treatment with this elicitor in the Garnacha variety produced a decrease in the concentration of the benzenoid compounds in the grapes and did not show any effect in Graciano [36]. These compounds are also involved in the aroma, highlighting 2-phenylethanol, with notes of rose, although it should be noted that its synthesis occurs mainly during fermentation [37].

Regarding alcohols, the following five compounds were identified in the musts: 1-heptanol, 1-octanol, 1-nonanol, 1-octen-3-ol and 2-ethyl-1-hexanol (Table 2).

As for the previous group, treatment with MeJ increased the concentration of these compounds, this effect being greater when applying the elicitor at post-veraison, except for 2-ethyl-1-hexanol, for which there were no differences between control and MeJ-treated samples. The results for total alcohols are shown in Figure 2d, where it is observed that the foliar application of MeJ enhanced their concentration and the moment of application has an influence in the same way that it has been described for the individual alcohols.

Table 2 shows the ten carbonyl compounds that were identified in the samples analysed. These compounds were: heptanal, (E)-2-heptenal, octanal, (E)-2-octenal, nonanal, (E)-2-nonenal, decanal, (E,E)-2,4-hexadienal, (E,E)-2,4-nonadienal and γ-decalactone. The highest concentration of (E)-2-heptenal and (E)-2-octenal was found in the post-veraison (MeJ-Post)-treated samples (Table 2). On the other hand, for octanal, decanal and γ-decalactone, an increase in their concentration was appreciated for samples after MeJ application, regardless of the time of application. In the case of (E)-2-nonenal, only the MeJ application at veraison increased its content. Furthermore, for heptanal, nonanal, (E,E)-2,4-hexadienal and (E,E)-2,4-nonadienal, no effect of MeJ application was observed (Table 2). Regarding total carbonyl compounds, their highest concentration was observed in the MeJ-Post samples; when applying MeJ at veraison, there was no effect on their content (Figure 2e).

Regarding C6 compounds, five were identified: hexanal, n-hexanol, (E)-2-hexenal, (E)-2-hexen-1-ol and (Z)-3-hexen-1-ol (Table 2). For this group of compounds, MeJ application at veraison (MeJ-Ver) enhanced the content of n-hexanol, (E)-2-hexen-1-ol and (E)-2-hexenal, whereas a decrease on (Z)-3-hexen-1-ol was observed when compared with control samples. For (E)-2-hexen-1-ol, a higher concentration was observed after the application of MeJ, being higher for MeJ-Ver; for (E)-2-hexenal, an increase in its content was appreciated after MeJ application, regardless of the time of application. In the case of (Z)-3-hexen-1-ol, its concentration decreased with the application of the elicitor, both at veraison and at post-veraison (Table 2). The total concentration of C6 compounds is shown in Figure 2f, which shows that the application of MeJ at veraison increased the content, while the post-veraison treatment did not affect the concentration of total C6 compounds. AS to the concentration of C6 compounds in general, it is highest during the pre-veraison and veraison stages but declines after veraison. It is possible that as long as the concentration of these compounds remains at a limit, the plant is able to synthesise them as a defensive response to the effect of the elicitor; however, once the concentration drops below this limit, the effect of the elicitor (post-veraison application) is diluted. This group of compounds in high concentrations can attribute negative aromas to the wines [38]; therefore, the treatment carried out at post-veraison would be more respectful since the content of these compounds in grapes did not increase compared to the control content, unlike the MeJ application at veraison (Figure 2f).

### 3.3. Effect of the Foliar MeJ Treatments on Grape Phenolic Compounds

Table 3 and Figure 2 show the results of grape phenolic composition in the control and in samples from treated grapevines with MeJ at veraison (MeJ-Ver) and post-veraison (MeJ-Post). The phenolic compounds identified and quantified in the Tempranillo Blanco grapes were five flavanols: quercetin-3-glucuronide (3-glcU), quercetin-3-glucoside (3-glc), kaempferol-3-galactoside (3-gal), kaempferol-3-glucoside (3-glc) and isorhamnetin-3-glucoside (3-glc); two flavanols: catechin and epicatechin; one hydroxybenzoic acid: gallic acid; five hydroxycinnamic acids: *trans*-caftaric acid, *trans*+*cis*-coutaric acids, caffeic acid, *p*-coumaric acid and *trans*-fertaric acid; and two stilbenes: *trans*-piceid and *trans*-resveratrol (Table 3).

Within the flavonols group, only the concentration of kaempferol-3-gal and isorhamnetin-3-glc in the grapes increased after the foliar application of MeJ, regardless of the time of application (Table 3). For the remaining compounds, quercetin-3-glc, quercetin-3-glcU and kaempferol-3-glc, there was no influence of the treatments in their content. Regarding the content of total flavonols (Figure 2g), only the application of MeJ at post-veraison increased their concentration in grapes. In general, flavonol synthesis occurs primarily during the early stages of fruit development and ends at around veraison; it is possible that at MeJ-Ver application, the plant had a sufficient concentration and did not need to synthesise more flavonols. However, at post-veraison time, the application of MeJ will activate its synthesis mechanism by increasing the concentration of these compounds. Quercetin-3-glc and quercetin-3-glcU were the most abundant phenolic compounds in Tempranillo Blanco grapes, as reported by other authors [39,40] for most white grape varieties. Flavonoid synthesis is conditioned by the expression of the enzyme flavanoid 3’5’-hydroxylase in white grape varieties, which limits the synthesis to quercetin, kaempferol and isorhamnetin compared to red grape varieties, where myricetin, laricitrin and syringetin are also synthesised [41]. Flavonols are yellow pigments that contribute to the colour of white varieties and provide several positive effects on human health due to their antioxidant activity [39,42].

With respect to flavanols, only catechin and epicatechin were found in the Tempranillo Blanco samples (Table 3). A significant increase in their concentration was observed in the treated samples (MeJ-Ver and MeJ-Post), regardless of the time of application. The results for total flavanols (Figure 2h) confirm that foliar treatment with MeJ produced an increase in the concentration of flavanols, this effect being independent of the time of application. These results contrast with those obtained by Portu et al. [8], where there was no effect of MeJ application in total flavanol content for the Tempranillo variety. This fact could be justified by the study by Ruiz-García et al. [43], where it was shown that flavanol accumulation after MeJ treatment depends on the grape variety. These phenolic compounds play a role in the grape and wine quality since they are responsible for sensory attributes, such as astringency, due to their ability to precipitate salivary proteins in the oral cavity. Moreover, flavanols are also involved in colour stability through co-pigmentation reactions [44].

In the group of hydroxybenzoic acids, only gallic acid was identified (Table 3), obtaining a higher concentration of this phenolic compound in MeJ grape samples, both at veraison and post-veraison, when compared with the content of control grapes. This result, as well as that of flavanols, contrasts with those obtained by Portu et al. [8] for the Tempranillo variety. Gallic acid has antioxidant and antifungal activities [45].

As for hydroxycinnamic acids, six compounds were identified: *trans*-caftaric acid, *trans+cis*-cutaric acids, caffeic acid, *p*-coumaric acid and *trans*-fertaric acid (Table 3). *trans*-caftaric and *trans*+*cis*-coutaric acids were the most abundant hydroxycinnamic acids in the grape samples; these compounds increased their concentration after MeJ application at post-veraison. However, the caffeic acid content was not affected by either of the two MeJ treatments; *p*-coumaric acid increased its concentration after MeJ application with respect to the control, regardless of the time of application. Finally, *trans*-fertaric acid content in grapes increased significantly after the foliar application of MeJ, with the highest concentration of this hydroxycinnamic acid being obtained for MeJ-Post. Figure 2i shows that the total concentration of hydroxycinnamic acids in the samples increased with both MeJ treatments, in agreement with Moro et al. [46], who observed higher concentrations of these compounds in juices after MeJ application. Hydroxycinnamic acids can act as precursors of vinylphenols during wine ageing in oak barrels [47] and, therefore, can be responsible for a depreciating wine sensory characteristic.

Finally, two stilbenes, *trans*-piceid and *trans*-resveratrol, were found in Tempranillo Blanco grapes. For these compounds, no significant differences in their concentration were obtained after MeJ foliar application (Table 3). However, it is observed that the total concentration of stilbenes was higher after the foliar application of MeJ (Figure 2j), regardless of the time of application. This result agrees with previous studies, such as those reported by Portu et al. [8,9], who observed that MeJ foliar application in Tempranillo plants improved stilbene synthesis. Hence, these results highlight the elicitor effect of MeJ since, although stilbene content varies among varieties, it is known that grapevine increases its synthesis in response to abiotic stresses [41], such as MeJ application or pathogen infections. Grapes and wines are among the major dietary sources of stilbenes for human nutrition [48], especially red varieties. These phenolic compounds have been demonstrated to possess a great range of biological activities potentially beneficial for human health, such as neuroprotective, antioxidant and antitumor effects, among others [49].

### 3.4. Influence of the Foliar MeJ Treatments on Must Nitrogen Compounds

Table 4 and Figure 2 show the results of must amino acid content in control and treated grapevines with MeJ at veraison (MeJ-Ver) and post-veraison (MeJ-Post). The most abundant amino acid in Tempranillo Blanco must was arginine (Arg), which, together with proline (Pro) and Gaba, represent approximately 76% of the total amino acids present in the must. This result coincides with that reported by Garde-Cerdán et al. [10], who observed that the most abundant amino acids in Tempranillo must were Arg, Pro and Gaba. In contrast, Gutiérrez-Gamboa et al. [50] reported that the most abundant amino acids in Tempranillo Blanco were arginine, glutamic acid, aspartic acid, citrulline and alanine.

The Tempranillo Blanco grape variety is an arginine-accumulating variety because the Pro/Arg ratio is less than 1. The data coincide with other white or red varieties [19,51]. However, Tempranillo Blanco differs from Tempranillo as Tempranillo tends to accumulate more proline than arginine [52].

It was observed that the foliar application of MeJ favoured the synthesis of aspartic acid, glutamic acid, valine, tryptophan, phenylalanine, isoleucine and leucine in the must, increasing their concentration in the samples, regardless of the time of application (Table 4). It should be noted that these amino acids are some of the main precursors of higher alcohols and esters [20]; therefore, they are the nitrogen sources that most influence the wine’s fermentative aroma [53]. The asparagine and histidine content also increased with the application of MeJ, but to a greater extent when it was applied at veraison or post-veraison, respectively, while the alanine concentration was only affected by the MeJ treatment carried out at veraison (Table 4). The content of the rest of the amino acids in the musts was not affected by either of the two treatments. In addition, foliar application of MeJ significantly increased the total amino acid content, with and without proline, with respect to the control, regardless of the time of application (Figure 2k,l), which could be of special interest for musts poor in nitrogen.

Garde-Cerdán et al. [10] observed that the foliar application of MeJ in Tempranillo increased the content of histidine, serine, tryptophan, phenylalanine, tyrosine, asparagine and methionine, although without affecting the total content of amino acids in the musts. However, Gil-Muñoz et al. [11] found that the application of MeJ increased the total amino acid content in the musts, affecting practically all the amino acids studied in grapes from the Monastrell variety. These results evidence the influence of the grape variety in response to the application of elicitors in vineyards [54].

## 4. Conclusions

In this work, the effect of the foliar application of MeJ to the vineyard, carried out at two phenological stages, veraison and post-veraison, on the phenolic, aromatic and nitrogen composition of Tempranillo Blanco grapes was studied for the first time. The results showed that, in general, the content of volatile compounds increased after MeJ treatments compared to control, mainly at post-veraison, such as total terpenoids, benzenoids, alcohols and carbonyl compounds. Generally, the increase in concentration for each of the groups of volatile compounds after treatment with MeJ was notable. Regarding phenolic compounds, their content increased in grape samples from MeJ foliar treatments. Furthermore, it was observed that the content of only hydroxybenzoic acid (gallic acid), flavanols and total stilbenes increased after treatment with MeJ, regardless of the time of application of this elicitor. Similarly, hydroxycinnamic acids improved their concentrations because of MeJ treatment, mainly after veraison. However, the influence of MeJ was less evident on flavonols. In terms of nitrogen composition, Tempranillo Blanco behaved as an arginine-accumulating variety, unlike its parent Tempranillo. In addition, foliar application of MeJ increased the content of different amino acids in the must in general, regardless of the time of application. Likewise, MeJ treatments significantly increased the content of total amino acids, with and without proline, with respect to the control, regardless of the time of application. Consequently, this study shows that both foliar applications of MeJ at veraison and post-veraison improved the content of different aromatic, phenolic and nitrogen compounds in Tempranillo Blanco grapes, with the best results being achieved with the post-veraison treatment. Therefore, the foliar application of MeJ treatment in Tempranillo Blanco vineyards seems to be a good tool to enhance grape quality. Further studies, with repeated years, should be considered as the next step.

## Figures and Tables

**Table 1:** General parameters in musts from control and MeJ treatments at veraison (MeJ-Ver) and post-veraison (MeJ-Post).

	Control	MeJ-Ver	MeJ-Post
Weight of 100 berries (g)	181.13 ± 15.04 ab	187.73 ± 9.85 b	161.50 ± 19.27 a
ºBrix	23.53 ± 1.31 a	22.33 ± 0.50 a	21.47 ± 2.06 a
Probable alcohol (% *v/v*)	13.82 ± 0.90 a	12.99 ± 0.34 a	12.41 ± 1.40 a
Glucose + Fructose (g/L)	241.61 ± 14.35 a	225.76 ± 0.93 a	214.58 ± 25.75 a
Glucose (g/L)	118.34 ± 7.14 b	111.18 ± 2.22 ab	103.31 ± 13.63 a
Fructose (g/L)	123.27 ± 7.38 a	122.92 ± 12.62 a	103.26 ± 25.26 a
pH	3.77 ± 0.09 a	3.77 ± 0.07 a	3.80 ± 0.06 a
Total acidity (g/L) *	4.89 ± 0.40 a	5.25 ± 0.34 a	5.01 ± 0.76 a
Tartaric acid (g/L)	6.83 ± 0.19 a	7.35 ± 0.93 a	6.44 ± 0.26 a
Malic acid (g/L)	2.44 ± 0.22 a	2.76 ± 0.41 a	2.77 ± 0.54 a
Total phenols (mg/L)	554.67 ± 50.15 a	546.97 ± 37.60 a	589.13 ± 26.34 a
Ammonium nitrogen (mg N/L)	93.34 ± 5.48 a	127.92 ± 31.29 b	106.08 ± 17.25 ab
Amino nitrogen (mg N/L)	164.85 ± 20.64 a	228.21 ± 39.60 b	220.00 ± 25.93 b
YAN (mg N/L)	258.19 ± 22.96 a	356.13 ± 70.89 b	326.08 ± 39.27 ab

* As g/L of tartaric acid. All parameters are listed with their standard deviation (*n* = 3). For each parameter, different letters indicate significant differences between the samples (*p* ≤ 0.05). YAN: yeast assimilable nitrogen.

**Table 2:** Benzenoid compound, alcohols, carbonyl compound and C6 compound concentrations (μg/L) in musts from control and MeJ treatments at veraison (MeJ-Ver) and post-veraison (MeJ-Post).

	Control	MeJ-Ver	MeJ-Post
***Benzenoid compounds***			
2-Phenylethanol	1.44 ± 0.23 a	8.48 ± 1.62 b	21.88 ± 2.12 c
2-Phenylethanal	10.48 ± 1.91 a	31.00 ± 2.29 b	39.28 ± 4.94 c
Benzyl alcohol	0.20 ± 0.03 a	0.55 ± 0.06 b	0.83 ± 0.08 c
***Alcohols***			
1-Heptanol	0.029 ± 0.004 a	0.084 ± 0.006 b	0.133 ± 0.014 c
1-Octanol	0.428 ± 0.090 a	0.647 ± 0.033 a	0.931 ± 0.196 b
1-Nonanol	0.104 ± 0.019 a	0.263 ± 0.026 b	0.355 ± 0.072 c
1-Octen-3-ol	0.579 ± 0.100 a	1.130 ± 0.039 b	1.863 ± 0.218 c
2-Ethyl-1-hexanol	0.619 ± 0.123 ab	0.479 ± 0.146 a	0.853 ± 0.145 b
***Carbonyl compounds***			
Heptanal	0.057 ± 0.010 a	0.057 ± 0.005 a	0.072 ± 0.012 a
(E)-2-Heptenal	0.135 ± 0.017 a	0.172 ± 0.016 b	0.272 ± 0.016 c
Octanal	0.032 ± 0.002 a	0.050 ± 0.006 b	0.054 ± 0.010 b
(E)-2-Octenal	0.190 ± 0.023 a	0.135 ± 0.020 a	0.802 ± 0.168 b
Nonanal	0.919 ± 0.168 a	0.792 ± 0.109 a	1.050 ± 0.171 a
(E)-2-Nonenal	0.112 ± 0.001 a	0.144 ± 0.020 b	0.123 ± 0.010 ab
Decanal	0.053 ± 0.008 a	0.089 ± 0.007 b	0.093 ± 0.017 b
(E,E)-2,4-Hexadienal	1.715 ± 0.193 a	1.818 ± 0.247 a	1.590 ± 0.197 a
(E,E)-2,4-Nonadienal	0.078 ± 0.014 a	0.090 ± 0.019 a	0.068 ± 0.014 a
γ-Decalactone	0.041 ± 0.013 a	0.067 ± 0.004 b	0.088 ± 0.018 b
***C6 compounds***			
Hexanal	87.73 ± 15.33 a	79.34 ± 2.46 a	77.77 ± 9.04 a
n-Hexanol	35.37 ± 6.24 a	55.71 ± 1.54 b	44.62 ± 5.26 a
(E)-2-Hexenal	30.24 ± 4.46 a	43.48 ± 1.70 b	43.29 ± 3.94 b
(E)-2-Hexen-1-ol	5.73 ± 1.03 a	16.07 ± 2.21 c	12.08 ± 1.72 b
(Z)-3-Hexen-1-ol	1.28 ± 0.20 c	0.85 ± 0.08 b	0.50 ± 0.10 a

All parameters are listed with their standard deviation (*n* = 3). For each compound, different letters indicate significant differences between the samples (*p* ≤ 0.05).

**Table 3:** Phenolic compounds’ concentration (mg/kg) in grapes from control and MeJ treatments at veraison (MeJ-Ver) and post-veraison (MeJ-Post).

	Control	MeJ-Ver	MeJ-Post
*Flavonols*			
Quercetin-3-glcU	71.79 ± 2.23 a	76.98 ± 15.88 a	94.42 ± 0.11 a
Quercetin-3-glc	98.58 ± 9.03 a	102.13 ± 18.80 a	122.94 ± 10.39 a
Kaempferol-3-gal	4.84 ± 0.24 a	6.05 ± 0.20 b	5.95 ± 0.21 b
Kaempferol-3-glc	17.07 ± 3.89 a	18.37 ± 0.11 a	18.04 ± 0.22 a
Isorhamnetin-3-glc	9.67 ± 1.28 a	14.85 ± 1.89 b	16.98 ± 2.47 b
*Flavanols*			
Catechin	23.45 ± 4.29 a	37.01 ± 3.23 b	36.82 ± 1.59 b
Epicatechin	22.40 ± 2.89 a	33.62 ± 3.24 b	39.85 ± 6.41 b
*Hydroxybenzoic acids*			
Gallic acid	11.04 ± 0.95 a	15.66 ± 1.32 b	17.12 ± 1.36 b
*Hydroxycinnamic acids*			
*trans*-Caftaric acid	17.17 ± 2.55 a	20.96 ± 2.16 ab	22.39 ± 1.44 b
*trans+cis*-Coutaric acids	11.28 ± 1.92 a	14.11 ± 0.56 ab	15.52 ± 1.07 b
Caffeic acid	0.11 ± 0.02 a	0.10 ± 0.02 a	0.09 ± 0.01 a
*p*-Coumaric acid	0.21 ± 0.04 a	0.34 ± 0.02 b	0.32 ± 0.03 b
*trans*-Fertaric acid	0.93 ± 0.01 a	1.03 ± 0.03 b	1.14 ± 0.03 c
*Stilbenes*			
*trans*-Piceid	0.37 ± 0.07 a	0.61 ± 0.13 a	0.55 ± 0.09 a
*trans*-Resveratrol	0.56 ± 0.14 a	0.57 ± 0.01 a	0.57 ± 0.03 a

Nomenclature abbreviations: gal, galactoside; glcU, glucuronide; glc, glucoside. All parameters are listed with their standard deviation (*n* = 3). For each compound, different letters indicate significant differences between the samples (*p* ≤ 0.05).

**Table 4:** Amino acid concentration (mg/L) in musts from control and MeJ treatments at veraison (MeJ-Ver) and post-veraison (MeJ-Post).

	Control	MeJ-Ver	MeJ-Post
Aspartic acid (Asp)	8.69 ± 1.83 a	15.01 ± 2.96 b	18.36 ± 1.57 b
Glutamic acid (Glu)	19.77 ± 1.34 a	29.03 ± 5.03 b	28.75 ± 3.00 b
Asparagine (Asn)	3.29 ± 0.02 a	7.62 ± 0.40 c	4.84 ± 0.72 b
Serine (Ser)	45.04 ± 4.12 a	51.47 ± 5.77 a	49.78 ± 2.69 a
Histidine (His)	7.63 ± 0.72 a	11.74 ± 1.45 b	17.47 ± 1.15 c
Glycine (Gly)	13.18 ± 2.91 a	12.88 ± 1.24 a	11.39 ± 1.08 a
Threonine (Thr)	49.11 ± 6.37 a	57.45 ± 4.48 a	58.26 ± 0.66 a
Citrulline (Cit)	10.46 ± 0.43 a	14.95 ± 2.86 a	13.99 ± 1.07 a
Arginine (Arg)	674.83 ± 67.56 a	751.42 ± 51.14 a	793.42 ± 63.75 a
Alanine (Ala)	53.13 ± 6.91 a	70.39 ± 7.61 b	61.81 ± 6.17 ab
γ-Aminobutyric acid (Gaba)	123.14 ± 11.37 a	121.39 ± 10.13 a	117.60 ± 13.98 a
Tyrosine (Tyr)	1.25 ± 0.26 a	1.57 ± 0.07 a	1.59 ± 0.31 a
Cysteine (Cys)	4.34 ± 0.73 a	5.36 ± 0.63 a	4.55 ± 0.41 a
Valine (Val)	26.46 ± 4.03 a	35.07 ± 3.23 b	39.62 ± 2.44 b
Methionine (Met)	6.08 ± 1.29 a	9.06 ± 0.06 a	8.81 ± 1.42 a
Tryptophan (Trp)	29.31 ± 4.01 a	35.59 ± 1.56 b	37.73 ± 0.66 b
Phenylalanine (Phe)	20.94 ± 2.01 a	39.00 ± 1.61 b	39.64 ± 3.49 b
Isoleucine (Ile)	12.14 ± 2.33 a	17.63 ± 1.39 b	19.35 ± 1.33 b
Ornithine (Orn)	5.65 ± 0.92 a	7.14 ± 1.77 a	5.81 ± 1.12 a
Leucine (Leu)	15.61 ± 2.06 a	28.22 ± 2.23 b	27.85 ± 2.68 b
Lysine (Lys)	1.63 ± 0.27 a	1.69 ± 0.12 a	1.94 ± 0.32 a
Proline (Pro)	257.22 ± 11.49 a	269.96 ± 26.55 a	276.81 ± 8.45 a

All parameters are listed with their standard deviation (*n* = 3). For each compound, different letters indicate significant differences between the samples (*p* ≤ 0.05).

## Data Availability

Data are contained within the article.
